# Inhibition of interferon gamma impairs induction of experimental epidermolysis bullosa acquisita

**DOI:** 10.3389/fimmu.2024.1343299

**Published:** 2024-05-10

**Authors:** Natalie Gross, Jana Marketon, Sadegh Mousavi, Kathrin Kalies, Ralf J. Ludwig, Katja Bieber

**Affiliations:** ^1^Lübeck Institute of Experimental Dermatology, University of Lübeck, Lübeck, Germany; ^2^Institute of Anatomy, University of Lübeck, Lübeck, Germany; ^3^Department of Dermatology, University Hospital Schleswig-Holstein Lübeck, Lübeck, Germany

**Keywords:** epidermolysis bullosa acquisita, treatment, neutrophils, model system, pemphigoid, IFN-γ

## Abstract

Epidermolysis bullosa acquisita (EBA) is a muco-cutaneous autoimmune disease characterized and caused by autoantibodies targeting type VII collagen (COL7). The treatment of EBA is notoriously difficult, with a median time to remission of 9 months. In preclinical EBA models, we previously discovered that depletion of regulatory T cells (Treg) enhances autoantibody-induced, neutrophil-mediated inflammation and blistering. Increased EBA severity in Treg-depleted mice was accompanied by an increased cutaneous expression of interferon gamma (IFN-γ). The functional relevance of IFN-γ in EBA pathogenesis had been unknown. Given that emapalumab, an anti-IFN-γ antibody, is approved for primary hemophagocytic lymphohistiocytosis patients, we sought to assess the therapeutic potential of IFN-γ inhibition in EBA. Specifically, we evaluated if IFN-γ inhibition has modulatory effects on skin inflammation in a pre-clinical EBA model, based on the transfer of COL7 antibodies into mice. Compared to isotype control antibody, anti-IFN-γ treatment significantly reduced clinical disease manifestation in experimental EBA. Clinical improvement was associated with a reduced dermal infiltrate, especially Ly6G+ neutrophils. On the molecular level, we noted few changes. Apart from reduced CXCL1 serum concentrations, which has been demonstrated to promote skin inflammation in EBA, the expression of cytokines was unaltered in the serum and skin following IFN-γ blockade. This validates IFN-γ as a potential therapeutic target in EBA, and possibly other diseases with a similar pathogenesis, such as bullous pemphigoid and mucous membrane pemphigoid.

## Introduction

Epidermolysis bullosa acquisita (EBA) is a chronic autoimmune disease that primarily affects the mucous membranes and/or the skin. It is distinguished and caused by the presence of autoantibodies that specifically target type VII collagen (COL7), a crucial protein responsible for maintaining the structural integrity of the skin’s basement membrane zone (1, 2). There is a clear and, so far, unmet medical need in the management of patients with EBA (3): First, EBA is notoriously difficult to treat. Despite combined immunosuppressive treatment, it takes on average 9 months to induce remission (4). Second, EBA patients are at an increased risk to develop other inflammatory diseases, such as lupus erythematosus and lichen planus, and metabolic and cardiovascular disease (5). Third, the overall risk of death in increased 2.5-fold compared to age- and sex- matched controls (6).

Use of preclinical model systems can be used to identify and validate new therapeutic targets. Using antibody transfer models of EBA or bullous pemphigoid (BP), a pemphigoid disease that shares several pathogenic pathways with EBA (7), we noted that depletion of regulatory T cells (Treg) led to a significantly aggravated inflammation and blistering in both model systems. On a molecular level, Treg depletion was accompanied by an increased expression of interferon gamma (IFN-γ), CXCL-9, IL-4, IL-13, and IL-10 in the skin of the mice (8). Except for IL-10, the impact of these afore mentioned cytokines on autoantibody-induced and neutrophil-mediated skin inflammation and blistering in EBA are unknown. Regarding IL-10, this cytokine inhibits complement-mediated neutrophil migration in the context of EBA and infection (9). One may assume that blockades of IL-4 and IL13 also have immunosuppressive effects because case reports showed a favorable response of the IL-4Ra antibody dupilumab in BP patients (10–12). On the other hand, IL4 may promote M2 macrophage proliferation (13), which would presumably lead to resolution of EBA or BP. We here focused on INF-γ because both recombinant protein and blocking antibodies are approved for the use in patients (14, 15).

There is no information on IFN-γ expression in EBA patients. In BP patients, decreased (16) or unaltered (17, 18) serum concentrations of IFN-γ have been reported. In the skin of BP patients, IFN-γ levels were identical to those of healthy controls (19), while in the blister fluid of BP patients, IFN-γ concentrations were higher compared to those in the serum of corresponding BP patients (18). Mechanistically, IFN-γ could impact on BP pathogenesis by modification of the expression hemidesmosomes on keratinocytes (20). A small-scale, open study addressed the impact of recombinant IFN-γ treatment in nine BP patients, all of which showed clinical improvement (14).

Taken together, there are missing or conflicting results regarding the expression and function of IFN-γ in EBA and BP. Based on our findings of an increased IFN-γ expression and a more severe phenotype following Treg depletion in experimental EBA, we hypothesized that inhibition of IFN-γ may dampen skin inflammation and blistering in EBA. To challenge this assumption, we employed the antibody transfer model of EBA.

## Materials and methods

### Animal experimentation

C57Bl/6J mice were originally obtained from the Jackson Laboratories (Bar Harbor, Maine, USA) and bred at the animal facility of the University of Lübeck, Germany. For the experiments, sex-matched mice between 8 weeks and 14 weeks of age were used. The mice were given standardized mouse chow and acidified drinking water provided *ad libitum* and maintained on a 12- h light/dark cycle. All clinical examinations, biopsies, and bleeding procedures were performed under anesthesia, using intraperitoneal (i.p.) administration of a ketamine (100 mg/g, Sigma-Aldrich, Taufkirchen, Germany) and xylazine (15 mg/g, Sigma-Aldrich) mixture unless otherwise specified. All animal experiments were conducted as per the European Community rules for animal care and were approved by the governmental administration [V242–12193/2020 (51-6/18)], Ministry for Energy, Agriculture, the Environmental and Rural Areas). Certified personnel performed the experiments.

### Generation of anti-mouse COL7^C^

Specific rabbit anti-mCOL7^C^ IgG from immune serum was isolated as previously described (21). In brief, New Zealandic white rabbits were immunized with 250 µg recombinant proteins of the non-collagenous (NC)-1 domain of murine COL7^C^ (mCOL7^C^) solved in complete Freund adjuvant. Boosters were administered three times every 14 days (250 µg, dissolved in incomplete Freund adjuvant). Serum samples were taken from the rabbits at 3-week intervals. Immunization and bleeding of rabbits were performed by Eurogentec (Seraing, Belgium). Total IgG from immune sera was purified by affinity chromatography using protein G. For isolation of specific rabbit anti-mCOL7^C^ IgG, a second affinity chromatography using mCOL7^C^-coupled Affi-Gel 10 (Bio-Rad, München, Germany) was performed using the manufacturer’s protocol with minor modifications: 5 mg/ml of mCOL7^C^ in MOPS buffer (0.1 M MOPS, 80 mM CaCl_2_, pH 7.5) was incubated with Affi-Gel 10 in MOPS buffer for 1 h at room temperature. Following incubation with MOPS buffer/ethanolamine (1:20) for 1 h, the mCOL7^C^-coupled gel was washed with PBS and prepared for use. For the isolation of mCOL7^C^-specific IgG, the rabbit anti-murine COL7^C^ IgG obtained from the first affinity chromatography was incubated under gentle shaking on the mCOL7^C^-coupled Affi-Gel 10 for 1 h at 4°C. The column was washed with PBS and subsequently with a washing buffer (0.1% Triton-X-100, 850 nM NaCl) until all nonspecific IgG was removed (OD280 < 0.01). To elute, the column was flushed with an elution buffer (0.1 M glycine, pH 2.8). The eluate was neutralized by Tris buffering at a pH of 7.2 and concentrated using an Amicon Ultra-15 filter (Millipore, Darmstadt, Germany). The reactivity of all IgG fractions was analyzed by immunofluorescence microscopy of murine skin.

### Induction of experimental EBA, randomization, and treatments

Antibody-induced transfer studies for inducing experimental EBA were performed following published protocols with minor alterations (21). The mice were given three i.p. injections of 150 µg of specific rabbit anti-mCOL7^C^ IgG on days 0, 2, and 4. Blocking murine IFN-γ was achieved by administering rat anti-murine IFN-γ (Hölzel Diagnostika Handels GmbH, Köln, Germany) i.p. at 125 µg, 250 µg, or 500 µg per mouse every other day starting 1 day before the first rabbit anti-mCOL7^C^ IgG injection and ending on day 13 (×8 total). As a control, a separate group of mice was injected with rat IgG1 isotype antibody (Hölzel Diagnostika Handels GmbH) at a dosage of 500 µg i.p. per mouse following the same injection schedule. Mice were randomly allocated to the groups in a sex-balanced ratio by an independent researcher. On days 4, 8, 12, and 15 (the final day), an investigator, unaware of the applied treatments, scored the affected areas of EBA by evaluating the presence of crust, erythema, lesions, and/or alopecia on individual body parts. The overall body score is calculated through a composite score of 2.5% for each ear, snout, and oral mucosa, 0.5% for each eye, 9% for the head and neck (excluding eyes, ears, oral mucosa, and snout), 5% for each front limb, 10% for the hind limb and tail, and 40% for the remaining trunk (22). The assessment of each mouse’s total burden included the evaluated EBA score, weight loss compared to the start of the experiment, changes in normal behavior, and clinical parameters such as body temperature or breathing frequency. Scoring was based on the severity of each parameter, with a score of 0 indicating no burden, 1 indicating a very slight change, 5 indicating a minor burden, 10 indicating a mild burden, and 20 indicating a severe burden. This was followed by an immediate early sacrifice of the individual mouse.

### Determination of secondary endpoints

Serum, ear skin, lesional, and non-lesional skin biopsies were obtained at the final day and were prepared for examination by histopathology, IF microscopy, ELISA, LegendPlex, and RT-PCR. For staining, sections from the ears were used, as they best reflect the individual EBA score of each mouse. For RT-PCR, however, similarly inflamed skin samples were taken to look for differences in equally inflamed lesions between the groups.

#### H&E staining of lesional skin

For histology, skin and ear samples were fixed in 3.7% paraformaldehyde and embedded in paraffin. Hematoxylin and eosin (H&E) staining was performed on 4–5- µm- thick sections following standard protocols. The dermal infiltration score was assessed by combining the individual epidermal thickness and split formation and infiltration of immune cells by an investigator unaware of the applied treatments.

#### Detection of tissue-bound IgG and C3

To detect tissue- bound rabbit IgG and murine C3, direct immunofluorescence microscopy was performed as described (21). Briefly, frozen sections were prepared from 6- µm- thick skin and ear sections and incubated with goat anti-rabbit antibodies reactive with rabbit IgG (Dako Deutschland GmbH, Hamburg, Germany) and murine C3 (MP Biomedicals LLC, Keyseberg, France). Both sections were labeled with fluorescein isothiocyanate (FITC), and the respective fluorescence intensity was evaluated by a blinded investigator using a semi-quantitative assessment method.

#### ELISA for detection of circulating total mouse and anti-rabbit IgG

Serum levels of circulating total mouse IgG and specific mouse anti-rabbit IgG were measured by ELISA using mouse IgG quantification kits (Bethyl, Montgomery, Texas, USA). In detail, each well was coated either with 100 µg affinity purified goat anti-mouse IgG-Fc coating antibody or 250 ng normal rabbit IgG (Bethyl). To reduce nonspecific binding, plates were blocked with 1% BSA in PBS-T at room temperature for 1 h. Sera were added with a prior dilution of either 1:32,000 for the detection of total IgG or 1:100 for anti-rabbit IgG. After incubation of 1 h, bound antibodies were detected by HRP-conjugated goat anti-mouse IgG (Bethyl) and tetramethylbenzidine (Thermo Fisher Scientific, Waltham, USA). The enzymatic color reaction was stopped by 2 M sulfuric acid (Carl Roth, Karlsruhe, Germany), and the change in OD was measured with a plate reader (Promega, Mannheim, Germany) at 450 nm. Standard reference curves were established using the provided mouse or rabbit sera.

#### Detection of cutaneous Ki-67, Ly6G, F4/80, CD4, and CXCL1 expression

In paraffin- embedded sections of lesional ear skin, proliferation, neutrophils, macrophages, and T helper cells, and CXCL1 were stained with the mAbs rat anti-mouse Ki-67 (Biolegend, San Diego, CA, USA), rat anti-mouse Ly6G (Biolegend), rabbit anti-mouse F4/80 (Abcam, Cambridge, United Kingdom), rabbit anti-mouse CD4 (Abcam), or rabbit anti-mouse CXCL1 (Invitrogen, Thermo Fisher Scientific, Waltham, MA, USA). Normal rat IgG (Biolegend) or rabbit IgG (Abcam) were utilized as isotype antibodies controls. The initial two stains were based on indirect immunofluorescence (IIF), while the latter three relied on immunohistochemistry (IHC). De-paraffinization of sections was done in histol (Carl Roth) and then re-hydrated in a descending ethanol series. To assess proliferation, macrophages, T helper cells, and CXCL1, two options were used: heat-induced antigen retrieval at 310°C under pressure in a 10 mM natrium citrate buffer (pH 6.0), or Tris-EDTA buffer [pH 9.0, composed of 10 mM Tris-base (SERVA Electrophoresis, Heidelberg, Germany), 1 mM EDTA v/v (Carl Roth)]. Antigen retrieval for detecting neutrophils staining was based on a 10-min-long enzyme digestion step that employed a ready-to-use pepsin solution (Thermo Fisher Scientific). To reduce nonspecific binding, blocking was performed with 5% goat normal serum (Dako, Glostrup, Denmark) or in a 1% BSA solution at room temperature for 20 –60 min. Primary antibody binding was performed either overnight at 4°C (neutrophils, macrophages, T-helper cells, and CXCL1) or for 90 min at room temperature (proliferation). The secondary antibody goat anti-rat Alexa Fluor 594 (Jackson Immuno Research, Ely, United Kingdom) was used for IIF staining, and the goat pAb to rabbit IgG (Abcam) was used for CD4 and CXCL1 IHC staining. After incubation for 1 h at room temperature, IIF stains were mounted with DAPI (Southern Biotech, Birmingham, Alabama, USA). CD4 detection was performed using the FastRed substrate kit (Abcam) or HIGHDEF red IHC chromogen (AP) kit (Enzo Life Science, Farmingdale, New York, USA) for CXCL1. Macrophage staining was performed with SignalStain Boost IHC Detection Reagent (HRP, rabbit) and SignalStain DAB Substrate Kit (both Cell Signaling Technology, Danvers, MA, USA) immediately after primary antibody binding according to the manufacturer’s instructions. All IHC stains were counterstained with Mayer’s hemalaun (Waldeck, Münster, Germany). Immunofluorescence and immunohistochemical staining were examined using a Keyence BZ-9000E series microscope (Osaka, Japan). The analysis immunohistochemical staining was conducted through light microscopy. The scoring system used for evaluation was quantitative and blinded, ranging from 0 to 4. A score of “0” indicated absence of staining in immune cells, while a score of “1” indicated staining of <10% of Ki-67, Ly6G, F4/80, and CXCL1 or one to five cells for CD4. A score of “2” denoted staining of between 11% and 30% or 6–10 cells, while “3” indicated staining of 31%–75% or 11–15 cells. Finally, a score of “4” represented staining of more than 75% or more than 15 positive cells.

#### Detection of serum cytokine concentration (Legendplex)

Different cytokine concentrations in the serum were analyzed using a custom-made LEGENDplex™ (Biolegend) as described by the manufacturer’s protocol. To analyze, cytokines comprised IL-1β, IL-4, IL-1α, IFN-γ, TNF-α, CXCL1, IL-10, IL-13, IL-17A, and GM-CSF.

#### Detection of marker expression in the skin by RT-PCR

To analyze gene expression in skin sections, 10 cryosections (12 µm) were prepared and used for RNA isolation, reverse transcription, and real-time RT-PCR as previously described (23). In short, total RNA was isolated from comparable inflamed lesional skin of both groups according to the manufacturer’s protocol (innuPrep RNA Mini Kit, Analytic Jena AG). After reverse transcription, the cDNA was added to either qPCR Master MixPlus or qPCR Master Mix SYBR Green Plus (Thermo Fisher Scientific) and amplified using an SDS ABI7900 system (Applied Biosystems, Darmstadt, Germany). The number of cDNA copies was normalized using the 2^ΔCT^ method with housekeeping gene GAPDH.

Markers selected for analysis were *Il17a* (Mm00439618_m1), *Itgam* (Mm00434455_m1), *Cxcl1* (Mm04207460_m1), *Ifngr1* (Mm00599890_m1), *Tnf* (Mm00443258_m1), *Il4ra* (Mm01275139_m1), *Csf2* (Mm00438328_m1), *Stat3* (Mm01219775_m1), *Il10* (Mm01288386_m1), *Stat6* (Mm01160477_m1), and *Gapdh* (Mm99999915_g1) all from Thermo Fisher Scientific.

### Statistical analysis

Data were analyzed using GraphPad Prism, version 9 (GraphPad Software Inc., Boston, MA, USA). Applied tests and confidence intervals are indicated in the respective text and figure legend. A p-value < 0.05 was considered statistically significant.

Sample size calculation for animal experiment were performed with SigmaPlot 12.0 (Systat Software Inc.) considering the percentage of with EBA affected body area as primary endpoint. The calculations assumed an expected standard deviation of 25% in the antibody transfer-induced EBA model, an alpha level of 5%, a power of 80%, and a minimum detectable difference from the positive control of 30%.

## Results

### Blockade of IFN-γ hinders induction of skin inflammation and blistering in antibody transfer-induced EBA

**Figure 1:**
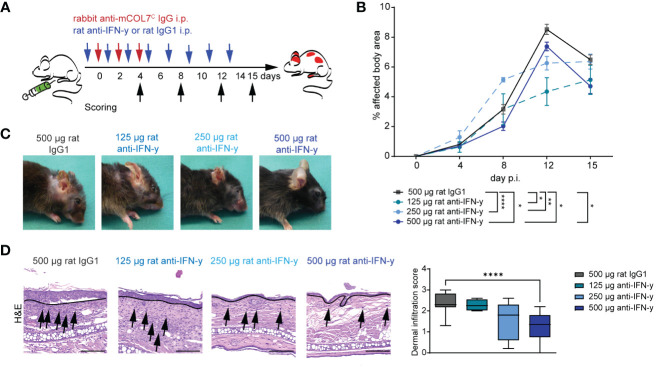
Blockade of IFN-γ dampens disease progression in experimental epidermolysis bullosa acquisita (EBA). **(A)** EBA was induced by repetitive intra-peritoneal (i.p.) injections of 3× 150 µg of specific rabbit anti-mCOL7^C^ IgG in C57BL/6 mice, and disease score was assessed on days 4, 8, 12, and 15. Murine IFN-γ was blocked by i.p. injection of a monoclonal rat anti- IFN-γ of either 125 µg, 250 µg, or 500 µg every other day starting 1 day prior to EBA induction. A control group was treated with a rat IgG1 isotype control following the same scheme. **(B, C)** Blockade of IFN-γ led to a significant lower skin inflammation in in all three treated groups on day 12. This effect persisted until day 15 for the highest used antibody concentration. Panel **(B)** displays the development of the with EBA- affected body surface area over the 15-day observation period. Statistical analysis: two-way ANOVA with Dunnett’s multiple comparison test (*p < 0.05, **p < 0.01, and ****p < 0.0001) with mean ± SEM and n = 5-15 per group. Panel **(C)** shows representative clinical pictures obtained on day 15 of the experiment. **(D)** Ears of mice after 15 days of experimental EBA were analyzed by hematoxylin and eosin staining (H&E) for dermal leukocyte infiltration. Mice treated with the highest anti- IFN-γ concentration showed a significantly lower dermal infiltration than the control group. Black arrows highlight some infiltrating cells, and the black line marks the dermal epidermal junction (DEJ). Statistical analysis: Kruskal–Wallis test with Dunn’s multiple comparison test (****p < 0.0001): data are presented as medians (black line), 25th/75th percentiles (boxes), and max/min values (error bars). Scale bar: 100 µm. p.i., post-EBA induction.

To assess the functional impact of IFN-γ inhibition in experimental EBA, we induced disease by transfer of COL7^C^ antibodies into C57Bl6/J mice that were treated with different doses of a function-blocking IFN-γ antibody or appropriate isotype control antibody (**Figure 1A**). In isotype antibody-treated mice, peak disease severity was reached on day 12 of the experiment with 8.5% of the body surface area affected by EBA skin lesion. Blockade of IFN-γ led to a significant lower EBA affected body area in in all three treated groups on day 12 with 4.3% (125 µg anti-IFN-γ), 6.3% (250 µg anti- IFN-γ), and 7.4% (500 µg anti-IFN-γ), respectively, whereas only the highest dose had already a significant effect on the disease score on day 8 of the experiment with 2.0% in the treated group and 3.2% in the control group. This effect persisted until day 15 with 4.7% of the affected body area in comparison to the control group with 6.5% (**Figures 1B, C**).

During the whole experiment, the total burden including the burden resulting from the affected body surface area by EBA skin lesions, weight loss, change in behavior, and general condition was assessed for each mouse. Mice treated with the highest treatment dose exhibited a significantly less total burden compared to the isotype antibody-treated control group on days 12 and 15, reflecting also the positive effect on the clinical disease score and the good tolerance of the treatment (**Table 1**).

**Table 1:** Total burden of mice of anti-IFN-γ- treated groups or control group in experimental epidermolysis bullosa acquisita over time.

Group	Number (f/m)	Mean total burden ± SEM				
		d0	d4	d8	d12	d15
500 µg rat IgG1	15 (8/7)	0.00 ± 0.00	3.40 ± 0.58	5.20 ± 0.88	8.73 ± 1.08	7.93 ± 0.60
125 µg rat anti-INF-y	5 (2/3)	0.00 ± 0.00	3.40 ± 1.08	5.40 ± 1.66	7.80 ± 2.20	9.40 ± 0.87
250 µg rat anti-INF-y	5 (2/3)	0.00 ± 0.00	2.80 ± 0.80	5.80 ± 1.43	10.20 ± 1.43	9.40 ± 0.87
500 µg rat anti-IFN-γ	16 (8/8)	0.00 ± 0.00	3.00 ± 0.53	2.63 ± 0.53	4.63 ± 0.75 (*)	4.75 ± 1.00 (*)

C57BL/6 mice were injected with 3× 150 µg rabbit anti-mCOL7^C^ IgG at days 0, 2, and 4. Murine IFN-γ was blocked by i.p. injection of a monoclonal rat anti- IFN-γ of either 125 µg, 250 µg, or 500 µg every other day starting 1 day prior to induction of epidermolysis bullosa acquisita (EBA). A control group was treated with a rat IgG1 isotype control following the same scheme. Total burden was assessed at days 0, 4, 8, 12, and 16 and comprises weight loss, change in normal behavior and clinical parameters, and disease score of EBA. Two-way ANOVA with Dunnett’s multiple comparison test, mean (± SED), n = 5–16. Significant differences in total burden compared to control group are indicated in gray.

**Figure 2:**
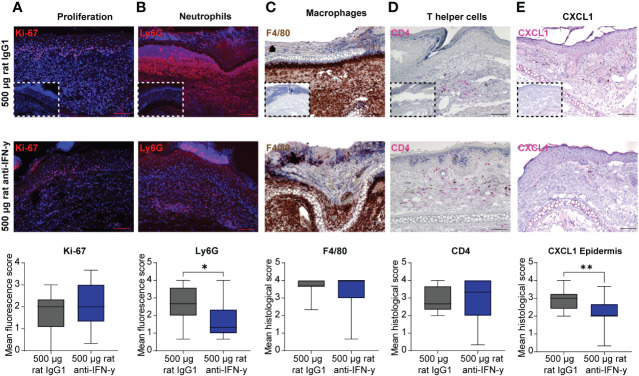
Dermal infiltration of Ly6G+ neutrophils and CXCL1 expression in the epidermis is decreased after blockage of IFN-γ in experimental epidermolysis bullosa acquisita (EBA). Lesional skin sections of day 15 of experimental EBA were stained for **(A)** proliferation (Ki-67), **(B)** neutrophils (Ly6G), **(C)** macrophages (F4/80), **(D)** T- helper cells (CD4), and **(E)** CXCL1 by **(A, B)** indirect immunofluorescence or **(C–E)** immunohistochemistry with n = 12–15 per group. Differences in proliferation, CXCL1, and the three immune cell populations were assessed between the control group (500 µg rat IgG1) and treated group with highest injected concentration of blocking IFN-γ antibody (500 µg rat anti- IFN-γ). The number of neutrophils in lesional skin and CXCL1 expression in the epidermis were significantly decreased in the treated group. **(A)** Proliferating cells (Ki-67+) and **(B)** neutrophils (Ly6G+) were detected by binding of a secondary antibody goat anti-rat Alexa Fluor 594 and are depicted in red. **(C)** Macrophages (F4/80+) were visualized by using DAB (brown cells), **(D)** T-helper cells (CD4+) and CXCL1 by using FastRed or HIGHDEF red chromogen (pink cells). Control staining with a **(A, B)** rat IgG or **(C–E)** rabbit IgG isotype antibody is depicted in small in the lower left corner of the control group of each staining. While no differences were noted for Ki-67, F4/80, and CD4, a significant decrease was documented for Ly6G+ cells and CXCL1 [Mann–Whitney U-test (*p < 0.05, **p < 0.01)]. Data are presented as medians (black line), 25th/75th percentiles (boxes), and max/min values (error bars). Scale bar: 100 µm.

The reduced clinical disease in mice treated with the highest dose of the IFN-γ antibody was accompanied with a reduced dermal leukocyte infiltration (**Figure 1D**). Further distinction of the dermal infiltrate showed that this was almost exclusively due to reduced numbers of Ly6G+ neutrophils, whereas the amount of F4/80+ macrophages and CD4+ T cells was identical between mice treated with isotype or anti-IFN-γ antibody (**Figures 2A–D**). Changes in the clinical phenotype were independent of keratinocyte proliferation (**Figure 2**), but a significant lower expression of CXCL1 in the epidermis in the treated group compared to the positive control group could be observed (**Figure 2E**). In contrast, there was no difference in CXCL1 levels in the dermis (data not shown).

**Figure 3:**
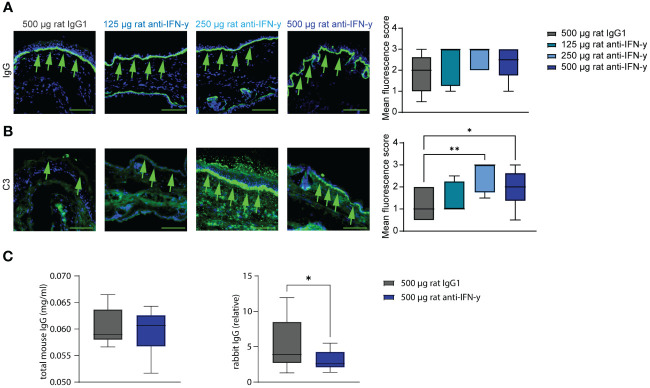
Blockade of IFN-γ increases C3 deposition in skin and decreases serum concentrations of COL7^c^ antibodies. Ears of mice of experimental EBA were analyzed for IgG and C3 deposition at the dermal epidermal junction (DEJ) at the end of experiment (green arrows). **(A)** No difference could be observed between the different groups regarding the IgG deposition. **(B)** Significant higher C3 levels were assessed in the two groups treated with 250 µg or 500 µg rat anti-IFN-γ compared to the control group. Statistical analysis: Kruskal–Wallis test with Dunn’s multiple comparison test with n = 5–10. **(C, D)** Total mouse and rabbit IgG in serum from mice of the control group and with 500 µg rat anti-IFN-γ- treated group were measured at the end of experimental EBA using ELISAs. There was no difference in levels of total mouse IgG, but significant lower levels of rabbit IgG in the treated group. Statistical analysis: Mann–Whitney U-test (*p < 0.05, **p < 0.01) with n = 15: data are presented as medians (black line), 25th/75th percentiles (boxes), and max/min values (error bars). Scale bar: 100 µm.

Tissue- bound IgG in the skin and circulating total mouse IgG were also identical among the groups. However, C3 deposits along the dermal epidermal junction were significantly increased in the two highest treated groups compared to that in isotype antibody- treated mice. Additionally in the highest treated group, significantly less of the injected COL7 antibodies could be found in the serum (**Figure 3**) with a possible dose dependency with increasing anti-IFN-γ antibody treatment (**Supplementary Figure S1**).

### IFN-γ inhibition has only marginal effects on systemic and local cytokine expression

To unravel the potential mode of action of the observed clinical effects of IFN-γ inhibition in experimental EBA, we assessed the systemic (serum) and local (skin) cytokine expression. Of note, of the 10 selected cytokines, only two showed significant differences when comparing isotype versus high-dose IFN-γ antibody- treated mice: Serum concentrations of IFN-γ increased, which is most likely due to increasing its’ half-life by the presence of the IFN-γ antibody. Serum concentrations of CXCL1 were significantly reduced in mice treated with IFN-γ antibody (**Table 2**).

**Table 2:** Analysis of inflammatory cytokines in the serum of anti-IFN-γ- treated mice or control mice after experimental epidermolysis bullosa acquisita.

Cytokine	500 µg isotype control	500 µg anti-IFN-y	*p-*value
IL-1β	8.02 ± 7.62	5.06 ± 5.53	0.3474
IL-4	2.06 ± 0.67	2.13 ± 0.84	0.9759
IL-1α	2.56 ± 1.73	2.66 ± 2.43	0.5765
IFN-y	0.60 ± 0.74	4.68 ± 4.93	0.0073
TNF-α	6.58 ± 3.86	7.68 ± 4.06	0.3705
CXCL1	154.65 ± 69.83	64.20 ± 47.57	0.0018
IL-10	9.47 ± 5.09	9.75 ± 4.56	0.7399
IL-13	2.90 ± 1.38	3.33 ± 2.45	0.9774
IL-17A	0.13 ± 0.25	0.94 ± 2.62	0.6513
GM-CSF	1.05 ± 0.83	1.31 ± 0.98	0.5555

Control mice (500 µg rat IgG1) or treated mice (500 µg rat anti-IFN-γ) were injected with rabbit anti-mCOL7^C^ IgG, and serum was taken for LEGENDplex™ cytokine analysis. Values are indicated as picograms per milliliter serum. Mann–Whitney U-test, mean (± SED), n = 11–12. Outliers were excluded from analysis. Significant differences in cytokine concentration are indicated in gray.

The expression of the corresponding cytokines (or their receptors) meanwhile showed no differences among mice treated with either isotype of function-blocking IFN-γ antibody in the skin (**Table 3**).

**Table 3:** Analysis of mRNA in skin biopsies of anti-IFN-γ- treated mice or control mice after experimental epidermolysis bullosa acquisita.

mRNA	500 µg rat IgG1	500 µg rat anti-IFNy	*p-*value
*IL-17A*	0.0001031 ± 0.0001340	0.00002909 ± 0.00003915	0.2704
*ITGAM*	0.5591 ± 0.5042	0.5949 ± 0.5219	0.7551
*Cxcl1*	0.09801 ± 0.1315	0.07746 ± 0.07282	0.9809
*IFNgR*	0.1120 ± 0.06405	0.1290 ± 0.07980	0.9809
*TNF*	0.5706 ± 0.7203	0.4785 ± 0.4238	> 0.9999
*IL4RA*	0.5537 ± 0.4584	0.9871 ± 1.252	0.6833
*Csf2*	0.009463 ± 0.007423	0.01555 ± 0.03054	0.548
*Stat3*	2.671 ± 1.880	3.018 ± 3.138	0.5164
*IL-10*	0.0008778 ± 0.0008235	0.001031 ± 0.001057	0.8088
*Stat6*	1.025 ± 0.5321	1.154 ± 1.071	0.7919

Control mice (500 µg rat IgG1) or treated mice (500 µg rat anti-IFN-γ) were injected with rabbit anti-mCOL7^C^ IgG, and lesional skin (of comparable disease level) was taken for mRNA extraction. Analysis of mRNA by qRT-PCR for the indicated markers was done relative to the housekeeping gene GAPDH using the 2^ΔCT^ method. Mann–Whitney U-test, mean (± SED), n = 12–15.

## Discussion

Depletion of Tregs in preclinical pemphigoid models that are based on antibody transfer into mice leads to aggravated clinical disease severity. This increase in clinical disease severity is accompanied by an increased expression of IFN-γ (8). We here aimed to elute if IFN-γ is of functional relevance in mediating autoantibody-induced tissue damage in experimental pemphigoid disease. Our results document that IFN-γ functions as a disease-promoting cytokine in antibody transfer-induced EBA.

This adds to our understanding of the contribution of cytokines to skin inflammation in this experimental pemphigoid disease, which has been continuously addressed since the determination of differential serum cytokine concentrations in the model (24). Most of the differentially expressed cytokines, like IFN-γ, have pro-inflammatory activities. More specifically, IL1β, IL17A, TNFα, GM-CSF, or CXCL1 promote autoantibody induced inflammation and blistering in antibody transfer models of EBA or BP (25–29). By contrast, CCL3/MIP1α had no impact on clinical EBA manifestation, despite elevated serum concentrations that highly correlated with disease severity (30). Of note, IL6, IL1ra and IL10 alleviated clinical disease manifestation in experimental models of EBA. In more detail, blockade of IL6 led to an aggravated disease in mice with experimental EBA, while treatment with recombinant IL6 led to a marked impairment of clinical EBA manifestation. Furthermore, blockade of IL6 led to reduced IL1ra serum concentrations, while recombinant IL6 increases IL1ra expression. Thus, IL6, by induction of IL1ra, counteracts the pro-inflammatory actions of IL1β (24). Blockade of IL10 was also associated with an aggravated disease manifestation in mice with immunization-induced EBA. Surprisingly, this effect was mediated through inhibition of neutrophil functions (9). With the results presented herein, we add to the understanding of the contribution of cytokines to EBA pathogenesis. More specifically, we identify IFN-γ as a cytokine that promotes cutaneous inflammation and blistering. Likewise, IFN-γ has also been shown to promote inflammation in mouse models of and/or patients with psoriasis (31), alopecia areata (32), and hyperinflammatory diseases, such as primary hemophagocytic lymphohistiocytosis, and several forms of secondary hemophagocytic lymphohistiocytosis, such as macrophage activation syndrome (33). Overall, this led to the approval of the IFN-γ antibody emapalumab for primary hemophagocytic lymphohistiocytosis (15). By contrast, in BP patients’ administration of recombinant IFN-γ led to a marked improvement of clinical disease severity in a small, open, single-center trial. Of note, clinical improvement was associated with a decrease in circulating antibody levels (14). Hence, IFN-γ could possibly modulate autoantibody production by shifting the presumed Th2-immune response in pemphigoid diseases more toward a Th1-immune response (34, 35). But with regard EBA, it could be shown that a Th1-like cytokine profile is needed to develop skin blisters, whereas the expression of Th2 cytokines protects from clinical manifestations (23), suggesting that blocking of IFN-γ in experimental EBA weakened the Th1-immune response and affected the observed lower affected body area. This, in turn, could have led to the observed reduced expression of CXCL1 in the epidermis, since keratinocytes produce higher levels during inflammation but decrease CXCL1 expression during wound healing (36–38). In addition, Hirose et al. (2013) have already shown that in experimental EBA, CXCL1 levels in lesional skin were significantly increased compared to control skin (25). As CXCL1 is one of the chemokines responsible for the chemoattraction of neutrophils (39), the migration of neutrophils was also reduced in the treated group. The discrepancy between the lack of effect on mRNA levels and the significant effect on protein levels for CXCL1 may be due to an initial upregulation of mRNA followed by a later increase in protein expression. However, we were unable to confirm this hypothesis as we did not collect sequential serum and skin biopsies. Additionally, it is important to keep in mind that samples for different experiments were taken from different sites, which could result in varying levels of expression regulation.

The serum concentrations of pathogenic anti-Col7 antibodies in mice treated with IFN-γ antibodies may be reduced due to indirect effects on the FcRn. Specifically, IFN-γ activates the JAK/STAT pathway, which downregulates the expression of the FcRn. This activation of the JAK/STAT-1 signaling pathway by IFN-γ can downregulate the functional expression of the MHC class I-related neonatal Fc receptor for IgG (40). Conversely, blocking IFN-γ would lead to an increase in FcRn expression. Therefore, the observed effects may also be partially attributed to the modulation of the half-life of pathogenic (auto)antibodies. (see also **Supplementary Figure S1**). The cause for the increased C3 deposits is potentially due to the IFN -γ-mediated locally increase in factor H (41). However, systemically, no difference in C3 or factor H could be observed (**Supplementary Figure S1**).

Regarding the mode of action of IFN-γ in promoting cutaneous inflammation and blistering in experimental EBA, we suggest that this is mainly mediated by an interaction of T cells and neutrophils. We previously demonstrated that T cells critically contribute to skin inflammation and blistering in experimental EBA through regulating neutrophil functions. NK and γδ T cells promote inflammation in EBA by promoting neutrophil recruitment. By contrast, Treg reduce inflammation in experimental EBA by altering the migratory capabilities of myeloid cells. Depletion of Treg aggravates skin inflammation in experimental pemphigoid disease (including EBA) and changes cytokine expression in the skin; with regard to IFN-γ, an increase is noted (8, 21). As T cells and macrophages are a main source of IFN-γ (42, 43), we assume that in the context of EBA, IFN-γ is locally produced in the skin by tissue-resident T cells and macrophages following the injection of anti-COL7^C^ IgG. In turn, this leads to an enhanced migration of neutrophils into the skin (8). This may be in part mediated by an increased expression of CD18 (44). In addition to promoting neutrophil extravasation into the skin, enhanced CD18 expression on neutrophils will also promote formation of the immunological synapse after binding to the tissue-bound immune complexes (45).

Regarding clinical translation of our findings, blockade of IFN-γ had a significant, but moderate effect on the clinical disease manifestation. This is in line with the observations made for blockade of other cytokines in experimental pemphigoid diseases (25–29). Thus, blockade of a single cytokine may not be sufficient to achieve clinically relevant effects in patients. Exceptions may be single patients, where pemphigoid disease is predominantly driven by a specific cytokine, i.e., by presence of certain polymorphism within specific cytokines or their receptors (46, 47). One possibility to overcome this limited therapeutic efficacy of blocking a singly cytokine in pemphigoid patients may be combined treatment. However, this would most likely be prone to an increased rate of adverse events. A more practicable and safe approach may be the modulation of signaling events following binding of cytokines to their receptors. Given the high level of promiscuity regarding receptor usage (48), effects of several cytokines are impaired following blockade of signaling downstream cytokine receptors. The potential clinical application of this approach is supported by observations in experimental models (49–53) and in case reports (54–56).

In summary, IFN-γ contributes to cutaneous inflammation and blistering in a preclinical model of EBA. The impact of IFN-γ inhibition on disease manifestation is significant but, compared to other drugs used in this model, relatively moderate. Thus, with the exception for personalized treatment approaches, blockade of signaling following cytokine binding to their respective receptors will more likely have an impact on patient morbidity.

## Data availability statement

The raw data supporting the conclusions of this article will be made available by the authors, without undue reservation.

## Ethics statement

The animal study was approved by Ministry for Energy, Agriculture, the Environmental and Rural Areas, Schleswig-Holstein, Germany. The study was conducted in accordance with the local legislation and institutional requirements.

## Author contributions

NG: Data curation, Investigation, Visualization, Writing – original draft, Formal analysis. JM: Investigation, Writing – review & editing, Data curation, Formal analysis, Visualization. SM: Writing – review & editing, Formal analysis, Investigation. KK: Writing – review & editing, Data curation. RL: Writing – original draft, Writing – review & editing, Conceptualization, Funding acquisition, Project administration, Resources, Supervision. KB: Writing – review & editing, Conceptualization, Data curation, Funding acquisition, Project administration, Supervision, Writing – original draft.
