# Supplementary material for: Inhibition of interferon gamma impairs induction of experimental epidermolysis bullosa acquisita
Source: Front Immunol. 2024 May 10;15:1343299. doi: 10.3389/fimmu.2024.1343299 (PMC11116581; doi:10.3389/fimmu.2024.1343299)
Supplement: Supplementary file 1 [file DataSheet_1.docx]

**Supplementary Materials and Methods**

***ELISA for detection of circulating C3 and complement factor H***

Serum levels of circulating C3 and complement factor H (CFH) were measured by ELISA using mouse C3 and CFH quantification kits (antibodies-online™, Limerick, Maine, USA) following manufacture’s protocols with a sample dilution of 1:25,000 for C3 and 1:50 for CFH.

**Supplementary Figure S1**


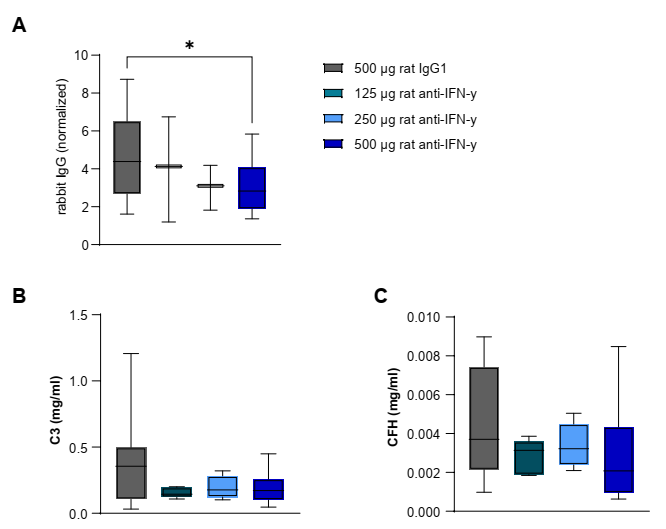


**Supplementary figure S1.** Supplementary figure S1. Blockade of IFN-y shows a dose dependency in reduction of rabbit IgG but no effect of C3 or CFH in serum. **(A)** Rabbit IgG in serum from mice of all groups were measured at the end of experimental EBA using ELISA. A dose dependency could be observed in lower serum levels of rabbit IgG with increased dosage of blocking rat anti-IFN-γ. With a significant reduction in the highest treated group compared to the control group. Statistical analysis: Mann-Whitney U-test (*p < 0.05). **(B and C)** Serum levels of C3 and CFH were measured in each group by ELISAs, but no difference could be observed. Data (n = 4-15) are presented as medians (black line), 25th/75th percentiles (boxes), and max/min values (error bars).
