# Supplementary material for: Inhibition of interferon gamma impairs induction of experimental epidermolysis bullosa acquisita
Source: Front Immunol. 2024 May 10;15:1343299. doi: 10.3389/fimmu.2024.1343299 (PMC11116581; doi:10.3389/fimmu.2024.1343299)
Supplement: Supplementary file 2 [file Table_1.docx]

Supplementary Material

Table S1. Summary of PERMANOVA for main and interaction effects of light environment and parental neighboring touch treatments on the multi-traits of parental and offspring ramets. The multi-traits of parental ramets include blade area, SLA, petiole length, SPL, aboveground biomass, root biomass, total biomass, R/S, LAR and LMR. The multi-traits of offspring ramets include total biomass, stolon biomass, leaf biomass, stolon biomass / leaf biomass, total blade area, number of ramets, length of the longest stolon, SSL of the longest stolon, LAR and LMR. Degrees of freedom followed by R^2^, F and *P* values are given for all the effects analyzed. Significant (*P* < 0.05) are shown in bold.

|  |  | Parental ramets | | | Offspring ramets | | |
| --- | --- | --- | --- | --- | --- | --- | --- |
| Source of variation | df | R^2^ | F | *P* | R^2^ | F | *P* |
| Fresh weight | 1 | 0.046 | 3.989 | **0.030** | 0.028 | 4.788 | **0.031** |
| Parental light environment (PL) | 1 | 0.297 | 26.054 | **0.001** | 0.519 | 89.260 | **0.001** |
| Offspring Light environment (OL) | 1 | 0.167 | 14.610 | **0.001** | 0.173 | 29.776 | **0.001** |
| Neighbor Touch (NT) | 1 | 0.068 | 5.970 | **0.008** | 0.048 | 8.317 | **0.005** |
| PL×OL | 1 | 0.071 | 6.208 | **0.006** | 0.036 | 6.262 | **0.009** |
| PL×NT | 1 | 0.075 | 6.557 | **0.004** | 0.032 | 5.494 | **0.015** |
| OL×NT | 1 | 0.005 | 0.473 | 0.658 | 0.019 | 3.301 | 0.058 |
| PL×OL×NT | 1 | 0.009 | 0.765 | 0.454 | 0.011 | 1.807 | 0.171 |
